# The COVID-19 Pandemic: A Pandemic of Lockdown Loneliness and the Role of Digital Technology

**DOI:** 10.2196/22287

**Published:** 2020-11-05

**Authors:** Syed Ghulam Sarwar Shah, David Nogueras, Hugo Cornelis van Woerden, Vasiliki Kiparoglou

**Affiliations:** 1 NIHR Oxford Biomedical Research Centre Oxford University Hospitals NHS Foundation Trust Oxford United Kingdom; 2 Radcliffe Department of Medicine University of Oxford Oxford United Kingdom; 3 EvZein Limited Oxford United Kingdom; 4 Public Health Agency Northern Ireland Belfast United Kingdom; 5 Division of Rural Health and Wellbeing University of the Highlands and Islands Inverness United Kingdom; 6 Institute of Nursing and Health Research Ulster University Belfast United Kingdom; 7 Nuffield Department of Primary Care Health Sciences University of Oxford Oxford United Kingdom

**Keywords:** COVID-19, coronavirus, pandemic, social isolation, loneliness, lockdown, social distancing, digital technology, social connectedness, social networking, online digital tools

## Abstract

The focus of this perspective is on lockdown loneliness, which we define as loneliness resulting from social disconnection as a result of enforced social distancing and lockdowns during the COVID-19 pandemic. We also explore the role of digital technology in tackling lockdown loneliness amid the pandemic. In this regard, we highlight and discuss a number of the key relevant issues: a description of lockdown loneliness, the burden of lockdown loneliness during the COVID-19 pandemic, characteristics of people who are more likely to be affected by lockdown loneliness, factors that could increase the risk of loneliness, lockdown loneliness as an important public health issue, tackling loneliness during the pandemic, digital technology tools for social connection and networking during the pandemic, assessment of digital technology tools from the end users’ perspectives, and access to and use of digital technology for tackling lockdown loneliness during the COVID-19 pandemic. We suggest that the most disadvantaged and vulnerable people who are more prone to lockdown loneliness are provided with access to digital technology so that they can connect socially with their loved ones and others; this could reduce loneliness resulting from social distancing and lockdowns during the COVID-19 crisis. Nonetheless, some key issues such as access to and knowledge of digital technology tools must be considered. In addition, the involvement of all key stakeholders (family and friends, social care providers, and clinicians and health allied professionals) should be ensured.

## Background

The COVID-19 pandemic has swept across the globe, resulting in about 29.3 million confirmed cases and about 0.93 million deaths worldwide as of September 15, 2020 [1]. The pandemic has compelled governments and authorities in affected countries to enforce preventive measures including enforced lockdowns, social distancing, self-isolation, and quarantine to slow down the spread of COVID-19 [2]. These preventative measures have contributed to social isolation and loneliness among people with specific characteristics [3,4]. The current COVID-19 crisis has profoundly affected social connections, and digital technology is playing an important role by providing virtual opportunities not only for businesses and health care delivery but also for social connection and networking. Numerous digital technology tools for social connection and business are available and being used by individuals according to their specific needs and requirements. However, certain groups of people are more disadvantaged because they do not have access to these tools and do not have the resources to get them. Hence, these underprivileged people are more likely to be socially disconnected and are at greater risk of loneliness, which is associated with serious adverse effects on social, physical, and mental health [4,5]. In this context, we identify and discuss a number of relevant and important issues as follows.

## What is Loneliness?

Loneliness is a subjective feeling of perceived “mismatch between the quantity and quality of social relationships” [6,7]. Loneliness is also commonly reported as a perceived discrepancy between the actual and desired social relationships of an individual [8].

A recent study on loneliness during the COVID-19 pandemic measured and reported loneliness as “chronic loneliness” (feeling lonely often or always) and “lockdown loneliness” (feeling lonely during the past 7 days) [3].

We consider lockdown loneliness more germane to, and of much interest during, the COVID-19 pandemic. We therefore focus on lockdown loneliness, which we define as “loneliness resulting because of social disconnection due to enforced social distancing and lockdowns during the COVID-19 pandemic and similar other emergency situations.”

## Burden of Lockdown Loneliness During the COVID-19 Pandemic

Although a loneliness epidemic [9] was reported in many countries (including Australia, the United Kingdom, and the United States) prior to the COVID-19 pandemic [10], the burden of loneliness has increased during pandemic lockdowns [11]. An increase in lockdown loneliness during the COVID-19 pandemic is evident from the latest statistics on coronavirus and loneliness in Great Britain, released in June 2020, which show that lockdown loneliness affected about 7.4 million adults (equivalent to about 14% of residents) during the COVID-19 pandemic lockdowns, while chronic loneliness remained at similar levels compared to prelockdown (2.6 million adults, equivalent to 5% of adults). However, about 80% of long-term lonely people were affected by lockdown loneliness during the pandemic [3].

The increase in loneliness during the COVID-19 pandemic has been attributed to increased social isolation because of lockdowns, social distancing, self-isolation, and quarantine measures aimed at reducing the spread of coronavirus [12]. The COVID-19 pandemic is therefore being labelled as the pandemic of loneliness [13,14].

## People More Likely to Be Affected by Lockdown Loneliness

The COVID-19 pandemic has not only resulted in disease-related illness and deaths but also has had serious adverse economic and sociopsychological impacts [13]. Lockdowns and social distancing during the COVID-19 pandemic have increased the risk of loneliness [11]. Recent studies showed that loneliness due to COVID-19 lockdowns, which we consider “lockdown loneliness,” is higher in adults who are single, divorced, separated, widowed, and/or living alone, as well as those individuals who have bad to very bad health [3]. In addition, lockdown loneliness has increased in young people (aged 16-25 years) [3,11] and seniors (>70 years old) [14]; however, older adults (55-69 years old) are reported to be less likely to be affected by lockdown loneliness [3].

In addition, the risk of loneliness in people of ethnic minority background has increased during COVID-19 lockdowns [12]. Fancourt et al [15] reported 35% higher loneliness in people of Black, Asian, and minority ethnic (BAME) origin compared to white British people (23% BAME versus 17% White), while a study by the British Red Cross reported about 12% higher prevalence of loneliness in BAME people compared to white people (46% BAME versus 41% White) during lockdowns [12]. Moreover, groups who were less likely to be affected by loneliness prior to the COVID-19 pandemic, such as families with young children, have also been affected by lockdown loneliness during the pandemic [12], but there is no statistically significant difference in lockdown loneliness and prelockdown loneliness in this group [3].

Risk of lockdown loneliness during the pandemic may be greater in people with limitations such as hearing loss [16], people who are digitally excluded [12], and those who are disconnected from colleagues because of working from home, which has been identified as a risk factor for loneliness [17].

## Factors Contributing to Lockdown Loneliness

The increase in loneliness during the pandemic has been attributed to COVID-19 lockdowns [3,11], during which social connection and social support become very limited because of the enforced physical distancing, social isolation, and quarantine measures [18]. These preventative measures have removed access to typical places used for social connection, interaction, and support [19], resulting in loneliness that could adversely affect physical and mental health and well-being [20]. In addition, COVID-19–related social distancing and isolation could result in sociopsychological harm, increasing the risk of loneliness in the most vulnerable and high-risk individuals, especially those who are socially, psychologically, and economically disadvantaged [19].

Loneliness is seldom observed in people who have social interaction, and socially active people have better overall health compared to those individuals who do not interact socially with others [21]. Loneliness is a social determinant of health [9] that is more commonly prevalent in people living in large cities [12] and areas that are deprived and geographically remote [22]. Other risk factors for loneliness include personal circumstances and characteristics, health and disability, and life transitions [23]. A higher risk of lockdown loneliness has been reported in females, younger people, and people who are dissatisfied with family, have negative self-perceptions about aging, have less contact with relatives, have the self-perception of being a burden on family and friends for support, listen to news related to COVID-19, have fewer resources for self-entertaining, and are digitally excluded [12,24].

## Lockdown Loneliness as an Important Public Health Issue

Loneliness is a major public health issue because it is associated with increased morbidity and mortality [4,5]. Loneliness is one of the key challenges that must be dealt with during the COVID-19 pandemic [25]. The situation could become more serious because levels of loneliness could rise due to an increase in the number of sociopsychological and mental health cases in the aftermath of the pandemic [18]. Empirical evidence shows that quarantine and lockdowns during viral infection epidemics, such as the SARS epidemic, result in more annoyance, fear, frustration, helplessness, isolation, loneliness, nervousness, sadness, and worry, and less happiness [26]. Similarly, the COVID-19 outbreak has resulted in psychological stressors related to the longer duration of quarantine, fear of infection, anxiety, feeling helpless, frustration, boredom, insufficient supplies, inadequate information, financial loss, and stigma, which further increase social isolation and loneliness [12,20,27]. At the same time, mental health and affective response to COVID-19’s threat to health are significantly associated with loneliness [28]. Moreover, the limited access to health care; social support (both formal and informal), interaction, and communication; economic, employment, and leisure opportunities; and other activities during the COVID-19 crisis has accelerated the risk of severe morbidity and mortality in high-risk individuals [12,29].

## Tackling Lockdown Loneliness

Tackling the rising tide of loneliness requires strengthening social connections and supporting people affected by lockdown loneliness during the COVID-19 crisis [18]. This requires efforts aimed at mitigating social isolation and facilitating social connectedness [30]. For tackling social isolation and loneliness during the COVID-19 pandemic, the World Health Organization has recommended maintaining social networks and staying connected with family, friends, colleagues, and community members via digital means [31]. More importantly, digital technology has become vital for addressing loneliness during the pandemic because other means of addressing loneliness (such as social prescribing) have become difficult if not impossible to access during the lockdowns. Even social prescribing for tackling loneliness has become digital social prescribing because it requires the use of digital technology during the pandemic [32].

## Digital Technology Tools for Social Connection During the COVID-19 Crisis

Digital technology is already a main feature of health systems and health and social care delivery [33], but its application has become critical during the COVID-19 pandemic [34]. Digital technology is enabling not only online and remote health consultations and a myriad of business activities but also connecting socially distant people during lockdowns and social distancing [35]. For example, digital technology enables online meetings, conferences, boardroom and team meetings, working from home [36], online teaching and learning, and even virtual cabinet meetings, which have all become almost the norm of daily life and business during the pandemic. Many technological companies, whether tech giants or start-ups, have either updated their existing portfolio of tools or developed new tools to fill the gap created by social distancing and lockdowns. A few examples of widely used online digital tools for social connection and networking include Zoom, Microsoft Teams, GoToMeetings, and Google Hangouts [37]. These tools are being used in developed [38] and developing countries [39] and have become more acceptable and widely adopted during the current pandemic. In addition, these virtual technologies are increasingly being used for providing social and cognitive support, supporting learning and teaching, enabling buying and selling, facilitating leisure and hobbies, and doing collaborative innovative research that can be done at a distance. It is expected that the use of these tools will increase and become a part of daily business in many fields, including health care, for a range of activities (eg, online medical consultations, treatment approaches, and interventions) [40].

More importantly, some companies are creating new products to help reduce loneliness and its impacts [41]. Two examples are the Spill online messaging app (an online mental health therapy platform) [42] and QuarantineChat, a one-on-one voice chat service that was developed to help people who are isolated during viral epidemics and emergencies beat boredom [43]. In addition, there are numerous other apps (eg, Headspace, Happify, and MindShift) that were developed to address mental health issues in general but could also be helpful in alleviating social isolation and loneliness during COVID-19 lockdowns [44].

## Assessment of Digital Technology Tools for Social Connectedness and Users’ Needs

A recent systematic review showed that a variety of digital tools, such as social media platforms, video conferencing, online voice and video networks, and social internet-based activities, are used for tackling loneliness in various settings [45]. These digital technology tools could be helpful in addressing lockdown loneliness during the COVID-19 pandemic. However, these tools must be assessed not only for their advantages but also for their limitations, including any negative impacts they may have on social relations, as the use of digital social media tools has been allegedly associated with the breakup of relationships and domestic abuse and violence in some families during quarantine and social isolation amid the pandemic [46,47]. It is also essential to evaluate how digital technology companies collect, manage, and use user data and whether there are any issues with regard to personal data security, privacy, and safety [48]. These issues are very important, especially for people who are more vulnerable, such as people with cancer [49], neurological conditions, and mental health problems, who could suffer more from the adverse impacts of the pandemic [31].

Therefore, digital technology tools used for health issues including lockdown loneliness must be safe, effective, and evidence-based [50]. The Anxiety and Depression Association of America has assessed and rated a number of apps from the end users’ perspective, including criteria such as effectiveness and ease of use [44]. Although most of these applications are for addressing mental health issues, they might be helpful in alleviating social isolation, loneliness, and mental health issues during COVID-19 lockdowns. However, before their adoption, these tools must be assessed for their accessibility, affordability, and acceptance by end users and patients [51].

## Access to and Use of Digital Technology for Combating Lockdown Loneliness

Combating lockdown loneliness during the COVID-19 pandemic requires changing the ways we connect socially [52], often through reliable, secure, easy to use, and effective digital technology tools [53]. More importantly, people who are most vulnerable to the adverse effects of the COVID-19 pandemic must not be digitally excluded [12]; rather, they should be actively provided access to digital technology [54]. Some people (eg, older adults) might have a low level of technological knowledge and literacy [55] and may therefore encounter difficulties and be less confident when using online digital technology tools [14]. Such people should be supported in developing their skills to effectively use these tools for social connection [12] to help alleviate lockdown loneliness during the pandemic. However, it is essential to consider and plan for the resolution of some other pertinent issues (eg, addressing the digital infrastructure [55], systems, and processes that may require development, upgrading, and investment) to support digital technology tools. The costs and maintenance of digital technological tools, and support and coordinated involvement of all key stakeholders (family and friends, social care providers, and clinicians and health allied professionals) are critical factors that must be taken into account to tackle lockdown loneliness.

## Conclusions

Digital technology has undoubtedly become critical for reducing and preventing social, physical, and psychological risks during the COVID-19 pandemic and addressing the short- and long-term impacts of social isolation and lockdown loneliness [18]. Nonetheless, most people affected by social isolation and lockdown loneliness during the pandemic might not feel lonely yet because these effects may take some time to show up [56]. It is therefore imperative that digital technology should not only provide tools to improve social connectedness and help in reducing lockdown loneliness but also enable people at risk of loneliness to take measures to avoid social isolation during the COVID-19 pandemic and in its aftermath. However, access to and costs and knowledge of digital technology tools are among the key issues that need urgent attention. Finally, tackling lockdown loneliness will require the active involvement of all key stakeholders that use these digital technology tools.

## Abbreviations

BAME: Black, Asian, and minority ethnic
